# Multiomic Integration Reveals Transposable Element–Embedded Regulatory Variants Underlying Brain Aging

**DOI:** 10.64898/2026.07.28.740948

**Published:** 2026-07-31

**Authors:** Sreejato Chatterjee, Shuangshuang Feng, Dean Zhang, Hongbo Liu

## Abstract

Genome-wide association studies (GWAS) have identified thousands of variants associated with brain aging, most in noncoding regions whose regulatory effects remain poorly understood. Transposable elements (TEs) constitute a major, understudied component of brain regulatory DNA. To interpret noncoding brain-aging variants through TE-derived regulatory architecture, we integrated brain-aging GWAS variants with TE annotations, ENCODE candidate *cis* -regulatory elements (cCREs), single-nucleus ATAC-seq accessibility maps, HOMER motif analysis, experimental enhancer–promoter interaction maps, and multiomic variant-to-gene resources. Of 6,323 genome-wide-significant variants, 633 (10.0%) localized within 367 TE-derived cCREs. These elements were predominantly enhancer-like and frequently carried transcription factor–associated annotation. Alu-derived elements formed the largest family component, followed by L1, L2, MIR and ERVL-MaLR elements. Single-nucleus accessibility maps resolved these variants across brain cell types, including a TcMar-Tigger–associated signal in oligodendrocytes, and motif analysis of SINE-associated cCREs identified reproducible nuclear-receptor, immune (PU.1:IRF8) and chromatin-regulatory (RFX-family) signatures. Because this localization reflects correlated variants within a limited number of independent loci rather than independent enrichment signals, we prioritized candidate regulatory variants and their target genes using the Brain Aging Genetic ScoreCard, which integrates 27 orthogonal evidence layers across independent, LD-defined loci and identified 25 loci with TE-derived regulatory support. The chromosome 17q21.31 *MAPT* locus emerged as the top candidate genome-wide, where a MIR-derived enhancer shows convergent evidence linking it to *MAPT* , a prioritization robust to locus-size effects. Overall, our study provides an integrative framework for interpreting noncoding GWAS variation through TE-derived regulatory architecture.

## Full Text Availability

The license terms selected by the author(s) for this preprint version do not permit archiving in PMC. The full text is available from the preprint server.

